# Plasma vascular endothelial growth factor B is elevated in non-alcoholic fatty liver disease patients and associated with blood pressure and renal dysfunction

**DOI:** 10.17179/excli2020-2647

**Published:** 2020-08-20

**Authors:** Xiaofeng Ye, Wen Kong, Mohammad Ishraq Zafar, Junchao Zeng, Rui Yang, Lu-Lu Chen

**Affiliations:** 1 Department of Endocrinology, Union Hospital, Tongji Medical College, Huazhong University of Science and Technology, Wuhan 430022, China; 2 Hubei provincial Clinical Research Center for Diabetes and Metabolic Disorders, Wuhan 430022, China; 3 Institute of Reproductive Health, Tongji Medical College, Huazhong University of Science and Technology, Wuhan 430030, China; 4 Healthcare Center, Union Hospital, Tongji Medical College, Huazhong University of Science and Technology, Wuhan 430022, China

**Keywords:** vascular endothelial growth factor B, non-alcoholic fatty liver disease, blood pressure, renal dysfunction, metabolism

## Abstract

Vascular endothelial growth factor B (VEGF-B) is a critical metabolic regulator in insulin resistance, and lipid distribution. We intended to ascertain the relationship between circulating VEGF-B and non-alcoholic fatty liver disease (NAFLD) in the general public. We recruited a total of 194 general participants for a routine physical health examination; of these, 84 participants were identified with NAFLD and 110 without NAFLD based on ultrasonographic findings. Homeostasis model assessment of insulin resistance (HOMA-IR), body mass index (BMI), HbA1c, liver function, kidney function, plasma VEGF-B levels and indexes of metabolic syndrome (blood pressure, fasting plasma glucose, fasting lipids) were evaluated. Plasma VEGF-B values were significantly higher in individuals with NAFLD compared to those without NAFLD (P = 0.022), and analysis of covariance confirmed this result. VEGF-B showed a positive correlation with γ-glutamyl transpeptidase (γ-GT) and HOMA-IR in univariate analysis (q = 0.242; P = 0.001; q =0.174; P = 0.019, respectively). Multiple linear regression analysis showed that γ-GT and ALT were independently correlated with VEGF-B even after adjusted for gender and age (q = 0.286; P = 0.01; q =0.237; P = 0.033, respectively). Moreover, plasma VEGF-B showed a powerful correlation with blood pressure and renal dysfunction. Plasma VEGF-B might be a new clinical variable related to NAFLD and could be a proper biomarker for the early detection of hypertension and renal dysfunction. However, further studies with large cohorts' size are warranted to validate our findings.

## Introduction

Vascular endothelial growth factors (VEGFs) are a family of signal proteins acting as important regulators of angiogenesis during physiological and pathological conditions (Olsson et al., 2006[30]; Zafar et al., 2018[41]). Vascular endothelial growth factor-B (VEGF-B) is a critical member of the VEGF protein family and is abundantly expressed in most tissues and organs (Aase et al., 1999[1]; Holmes and Zachary 2005[15]; Li et al., 2001[22], 2012[23]). Traditional studies on VEGF-B largely concentrated on their effects on neurotrophy, angiogenesis, and neuroprotection, while some ruled out the association between VEGF-B overexpression and tumor growth, invasion, diabetic retinopathy and others (Abedin et al., 2010[2]; Falk et al., 2009[9]; Olofsson et al., 1996[29]; Zhong et al., 2011[43]). Few recent studies identified that VEGF-B is tightly related to metabolism and obesity (Gomez-Ambrosi et al., 2010[12]; Hagberg et al., 2010[13], 2012[14]). Another study found that the overexpression of VEGF-B in the mouse heart is related to the increase of ceramide and triglycerides, which led to cardiac hypertrophy (Karpanen et al., 2008[16]). Chen et al. reported that adipose VEGF-B repression induced adipose tissues transferred toward white adipose for energy storage, and the glucose metabolism and lipid metabolism are broadly changed (Chen et al., 2020[5]). 

There are limited clinical data which reported the pathological roles of VEGF-B in obese subjects and those with metabolic syndrome. Gomez-Ambrosi et al. reported that the serum VEGF-B levels were significant higher in the obese group compared with the lean group (Gomez-Ambrosi et al., 2010[12]). Sun et al. compared the concentration of VEGF-B between individuals with type 2 diabetes (T2DM) and healthy controls and highlighted that there was no significant difference between the two groups (Sun et al., 2014[38]). However, the findings in this study indicated that the use of thiazolidinediones can influence the circulating VEGF-B levels (Sun et al., 2014[38]). Cheng et al. found that individuals with polycystic ovary syndrome (PCOS) had higher circulating VEGF-B levels than the age-matched healthy controls (Cheng et al., 2016[6]). Another study reported that plasma VEGF-B elevated in newly diagnosed T2DM and impaired glucose regulation (IGR) patients, compared with healthy controls (Wu et al., 2017[40]). These studies drew a substantial association between VEGF-B and insulin resistance (IR), T2DM. 

Clinical and preclinical studies have demonstrated IR as one of the core predisposing factors for metabolic diseases like obesity, T2DM, PCOS, and non-alcoholic fatty liver disease (NAFLD) (Eckel et al., 2005[8]; Kitade et al., 2017[18]; Macut et al., 2016[25]). Up to now, there are no existing data on circulating VEGF-B levels in NAFLD individuals. Therefore, in this study, we intended to investigate and compare the plasma VEGF-B levels between cohorts with NAFLD and without NAFLD. Additionally, we also aimed to investigate relationships between plasma VEGF-B level and blood pressure (BP), kidney function, glucose, and lipid metabolism.

## Materials and Methods

### Cohorts

A total of eighty-four cohorts (60 males, 24 females), aged 18-70 years, with fatty liver diagnosed by ultrasound, were recruited in the NAFLD group in this study. Whereas, one hundred and two adult individuals (49 males and 53 females), aged 18-70 years without any ultrasound evidence of NAFLD, were recruited in the control group. These cohorts attended the Healthcare Center of Union Hospital of Tongji Medical College, Huazhong University of Science and Technology for a routine physical health examination between May 2017 and August 2017. The cohorts were recruited if they met following inclusion criteria: negative tests for the presence of hepatitis B surface antigen and antibody to hepatitis C virus; no history of current or past excessive alcohol drinking (a threshold of < 20 g/d for women and < 30 g/d for men); no active or previous history of liver cirrhosis and other chronic liver diseases; no active history insulin treatment; and female participant not to be pregnant at the time of recruitment. The cohorts undertook a complete health examination, including anthropometric measurements (body weight, height), BP measurement, fasting plasma glucose (FPG), HbA1c, blood lipids, fasting insulin, liver function test, and kidney function tests. HOMA-IR was calculated as [fasting insulin (μU/mL) ×FPG (mmol/L)/22.5]; and BMI was calculated using the following formula: [weight (kg)/ height² (m)]. Hypertension was defined as systolic blood pressure (SBP) > 140 mmHg, or diastolic blood pressure (DBP) > 90 mmHg.

The Ethical Committee of Tongji Medical College, Huazhong University of Science and Technology approved the current study, and our study was accordant with the declaration of Helsinki. We got the written informed consent from each participant before recruited them to the study.

### VEGF-B assay

The circulating VEGF-B levels were tested by a commercially available human VEGF-B ELISA kit (USCN Science Co, Wuhan, China) in accordance with the manufacturer's protocol. The detection range of the VEGF-B ELISA test was 15.6 to 1000 pg/mL, and intra-assay and inter-assay variations were < 10 % and < 12 %, respectively. The detection limit of this ELISA kit is 5.5 pg/mL.

### Determination of fatty liver

The fatty liver was determined using ultrasonography by a proficient radiologist with extensive experience in abdominal ultrasound examinations using a high-resolution B-mode topographic ultrasound system with a 3.5 MHz probe (HDI 5000, Philips, Bothell, WA, USA). The fatty liver ultrasonography assessment consisted of at least two of the following findings: diffusely increased echogenicity ('bright') liver with liver echogenicity higher than kidney or spleen, deep attenuation of ultrasound signal, and vascular blurring (Farrell et al., 2007[11]).

### Statistical analysis

We used Statistical Package for Social Sciences, version 22.0 (SPSS, Chicago, IL, USA) to analyze the data. Results were shown as mean ± SD. The differences of continuous variables between the NAFLD group and the control group, the hypertension group and the control group were analyzed using t-tests. We adjusted age, gender and FPG to perform the analysis of covariance (ANCOVA) between the NAFLD group and the control group. We analyzed the relationship between variables by Spearman's correlation coefficient test. Multivariate analysis was performed between sex, age, BMI, liver function, kidney function, HOMA-IR, HbA1c, blood lipids, BP and VEGF-B. All P values presented are two-tailed, and values less than 0.05 are recognized as statistically significant.

## Results

**Figure 1:**
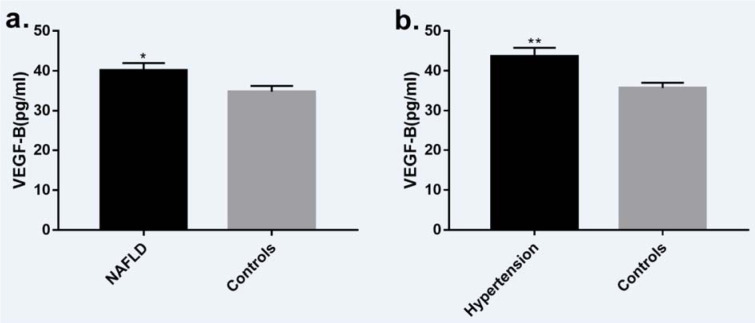
a. Plasma VEGF-B concentration was significantly higher (P = 0.022) in subjects with NAFLD (n=84) compared with the control group (n=102). b. Plasma VEGF-B concentration was significantly higher (P = 0.003) in subjects with hypertension (n=53) compared with the control group (n=141). Data presented as mean ± SEM. NAFLD: non-alcoholic fatty liver disease

The mean age of the cohorts was 48.77 ± 11.01 years. Of all cohorts, 58.2 % were male (n = 113), and 41.8 % were female (n = 81). The average BMI recorded was 25.44 ± 4.79 kg/m^2^, and 24 of them had diabetes. The cohort group allocation was based on medical history and liver ultrasonography, and the entire cohort was divided into groups, of these 102 patients (52.58 %) without NAFLD in the control group, and 84 patients (47.42 %) those have NAFLD (most of them presented mild liver lipid accumulation) in the NAFLD group. The clinical characteristics of these groups are displayed in Table 1(Tab. 1). Briefly, age, estimated glomerular filtration rate (eGFR), alkaline phosphatase (ALP), and total cholesterol (TC) did not show a significant difference. Instead, FPG, HOMA-IR, BP, LDL-C, triglycerides (TG), BMI, alanine transaminase (ALT), aspartate aminotransferase (AST), γ-glutamyl transpeptidase (γ-GT), uric acid (UA) and HbA1c were all significantly higher in the cohorts of the NAFLD group, and levels of HDL-C were significantly lower in the NAFLD group. The VEGF-B measured values were significantly higher (P = 0.022) in NAFLD individuals compared to non-NAFLD subjects (Figure 1a(Fig. 1)). Analysis of covariance between the two groups also indicated that VEGF-B were higher (p=0.047) in subjects with NAFLD after adjusting for FPG, gender and age. The VEGF-B measured values were significantly higher (P = 0.003) in hypertension individuals compared to control subjects (Figure 1b(Fig. 1)). 

We analyzed the correlation between VEGF-B and the other variables of the two groups by the Spearman correlation coefficient test. Our results revealed a positive correlation between γ-GT (q = 0.242; P = 0.001), HOMA-IR (q =0.174; P = 0.019), SBP, DBP), UA, creatinine (Cr), cystatin C (CysC), TG and VEGF-B. Whereas, we also found a negative correlation between eGFR (q = -0.185; P = 0.01), HDL-C and VEGF-B. The variables like FPG, HbA1c, BMI, age, ALT, AST, ALP, TC, LDL-C did not show any correlation with VEGF-B level (Supplementary Table 1).

In multivariate regression analysis, we adjusted gender and age to determine the associations between VEGF-B and selected covariates (BMI, liver function, kidney function, HOMA-IR, HbA1c, blood lipids and BP) (Table 2(Tab. 2)), we found that γ-GT and ALT were independently correlated with VEGF-B (q = 0.286; P = 0.01; q =0.237; P = 0.033, respectively). Besides, we also identified that SBP and DBP were correlated with VEGF-B (q = 0.345; P = 0.002; q =0.284; P = 0.01, respectively), and the eGFR (q = -0.17, P = 0.02) and other kidney function indexes (UA, Cr, CysC) were also correlated to VEGF-B. Additionally, our results indicated a significant inverse correlation between VEGF-B and HDL-C (q = -0.273; P = 0.014).

See also the Supplementary data 1 and 2.

## Discussion

Fatty accumulation within the liver origin from metabolic, and it is one of the main reasons of liver diseases (Pappachan et al., 2014[31]), with a predicted prevalence of approximately 25 % in the general population (Satapathy and Sanyal, 2015[35]). NAFLD includes a broad clinical and histological spectrum extending from simple hepatic steatosis to non-alcoholic steatohepatitis, with varying degrees of inflammation and fibrosis, which could potentially lead to cirrhosis. Fatty infiltration in the liver is a critical member of the metabolic disorders, NAFLD individuals is similar to altered body fat distribution individuals, who are at higher risk of cardiovascular disease and diabetes (Kelly et al., 2009[17]; Kotronen and Yki-Jarvinen, 2008[19]; Mariani et al., 2013[26]; Pappachan et al., 2014[31]; Pisto et al., 2014[33]; Sookoian et al., 2011[37]). 

There are no specific biomarkers of NAFLD. Recently, the essential roles in lipid metabolism, insulin resistance, and adverse influence on diabetes and metabolic disorders have been attributed to VEGF-B (Hagberg et al., 2010[13], 2012[14]). Mammalian VEGF-B intervenes in maintaining energy balance (Bry et al., 2014[4]; Zafar et al., 2017[42]). By regulating lipid delivery to fat-burning tissues, VEGF-B coordinately increased ectopic lipid deposition, decreased muscle glucose uptake, and caused hyperglycemia (Hagberg et al., 2010[13], 2012[14]).

This is the first study to attempt to measure the circulating VEGF-B in NAFLD individuals and to figure out if VEGF-B could be a predictor of NAFLD. The primary finding of our study is that the mean plasma values of VEGF-B in individuals with NAFLD were significantly higher compared with the control cohorts without NAFLD. Also, Pearson's correlation analysis revealed that circulating VEGF-B levels is positively associated with γ-GT. Besides, gender and age-adjustment, multivariate regression analyses showed γ-GT and ALT were independently correlated with VEGF-B. These results demonstrate that VEGF-B plays a significant role in NAFLD. Our findings are identical with the evidence that inhibition of VEGF-B signaling pathways or decreased expression of VEGF-B relieves excess fatty accumulation in the liver, and normalizes glucose levels, and ameliorates dyslipidemia (Hagberg et al., 2012[14]). Furthermore, an animal study suggested that VEGF-B expression was associated with liver cirrhosis (Ujiie et al., 2020[39]). These studies confirmed that VEGF-B has a vital role in the pathogenesis of NAFLD.

Interestingly, the present results displayed positive relations between circulating VEGF-B concentrations and UA, Cr, and CysC, and negative relation between circulating VEGF-B concentrations and eGFR. VEGF-B expresses in human kidneys and is considered to have critical pathological roles in developing diabetic nephropathy (Falkevall et al., 2017[10]; Lagercrantz et al., 1998[20]). It was reported that patients with macro-albuminuria had higher VEGF-B levels than those with non-albuminuria and microalbuminuria in T2DM (Sun et al., 2014[38]). Moreover, clinical evidence showed that diabetic kidney disease (DKD) patients had elevated VEGF-B levels in the kidney (Falkevall et al., 2017[10]). These consequences demonstrated that VEGF-B reverberated the severeness of diabetic nephropathy and that it probably is an advisable biomarker for the discovery of diabetic nephropathy. Falkevall et al. conducted experiments in DKD mice and showed that renal VEGF-B expression is positive, which is related to the severity of the disease. Inhibition of VEGF-B signal transduction in DKD mice attenuates renal lipotoxicity, resensitizes podocytes to insulin signal transduction, which hinders the progression of DKD-associated pathologies, and leads to the prevention of renal dysfunction (Falkevall et al., 2017[10]). Our study provided evidence that VEGF-B may also play a role in the pathogenic of kidney disease in normoglycemic subjects. However, the mechanism that VEGF-B acts in renal diseases in normoglycemic subjects is unclear. Further studies are warranted to explain the precise part of VEGF-B in normoglycemic subjects and fatty liver patients.

Furthermore, the mean plasma values of VEGF-B in individuals with hypertension were significantly higher compared with the control cohorts without hypertension, and we found a robust correlation between plasma VEGF-B levels and SBP, DBP. The underlying mechanism that VEGF-B effects on hypertension can partly be explained by a most recent animal study (Zhu et al., 2020[44]). In this study, the author revealed that VEGF-B participates in the progression of obesity-associated hypertension in two mouse models of obesity, and they described that inhibition of VEGF-B may have benefit for the treatment of obesity-associated hypertension (Zhu et al., 2020[44]). As most cohorts in our study had a normal BP range, we suggest that plasma VEGF-B might be a considerable biomarker for the early recognition of hypertension.

Although the exact mechanisms associated with the onset of NAFLD remain uncovered, it has been agreed that IR is a major contributor for the progression of NAFLD (Birkenfeld and Shulman, 2014[3]; Lee et al., 1998[21]; Machado and Cortez-Pinto, 2005[24]). In the present study, Pearson's correlation analysis confirmed that VEGF-B has a significant relationship with HOMA-IR (r=0.174, P=0.019). Similarly, studies in newly diagnosed T2DM and PCOS patients indicated that circulating VEGF-B concentrations were positively correlated with IR (Cheng et al., 2016[6]; Wu et al., 2017[40]). Besides, Sun et al. discovered that in T2DM patients the circulating VEGF-B levels decreased when treated with thiazolidinediones, which can inhibit peroxisome proliferators-activated receptor-γ activity and result in insulinresistant decrease (Sun et al., 2014[38]). However, multivariate analysis shows that the relation between VEGF-B and HOMA-IR is insignificant. How VEGF-B effects on insulin resistance in NAFLD subjects remain poorly understood, however, animal studies had provided some hints. The endothelial cell-mediated lipid uptake is regulated by VEGF-B and the downstream signaling pathway (Hagberg et al., 2010[13]). And the excess lipid deposition in tissues like skeletal muscle and liver will cause insulin resistance and disrupts the metabolism of nutrients (Perseghin et al., 1999[32]; Samuel et al., 2010[34]). However, the liver absorbs nutrients through the liver sinusoids, in which the permeability is different from endothelial cells, which may be the reason for our multivariate analysis result. No specific study on the relationship between VEGF-B expression levels and insulin resistance in NAFLD has been reported, so we cannot confirm that the underlying mechanism that VEGF-B triggers insulin resistance in NAFLD is identical to the mechanism that VEGF-B triggers in other insulin resistance disorders.

VEGF-B takes part in the glucose homeostasis in various aspects, including pancreatic b-cells function, insulin secretion, and insulin resistance in T2DM (Hagberg et al., 2012[14]; Ning et al., 2020[27]; Wu et al., 2017[40]). However, our data show that there is no correlation between VEGF-B and HbA1c and FPG. In contrast, some authors have reported that VEGF-B levels are positively correlated with HbA1c in newly diagnosis T2DM patients and PCOS patients (Cheng et al., 2016[6]; Wu et al., 2017[40]). Whether these discrepancies depend on the different patient group or are due to the different sample sizes is not for sure and needs further exploration. It is needed to consider that the subjects involved in the present study were included in the healthcare center, and our population had no particular diseases and had no special treatment. 

As an important portion of metabolic syndrome, hyperlipidemia is correlated with VEGF-B, too. An animal study found that vegfb knockout was related to a decrease in plasma triglyceride and LDL and an increase in HDL levels in diabetic mice (Hagberg et al., 2012[14]). Furthermore, Wu et al. suggested that higher VEGF-B levels were correlated to higher blood TG, which indicated that VEGF-B probably affects blood lipids in humans as well (Wu et al., 2017[40]). In our study, blood TG, HDL-C and LDL-C were significantly different between fatty liver individuals and control individuals, and the correlation analyses indicated that plasma VEGF-B levels were significantly associated with blood TG and HDL-C, and multivariate analysis showed that VEGF-B had an inverse correlation with HDL-C, which is identical with the animal research (Hagberg et al., 2012[14]). 

We admit that there are some limitations in this study. First, our study was a cross-sectional study, so we couldn't know the causal relationship between VEGF-B and the evaluated covariates. A prospective study with clinical intervention will be a recommended solution to clarify this point. Second, our study had a relatively small sample size, and it is recommended to confirm our results in larger cohorts. Third, although liver ultrasound is accurate to diagnose NAFLD, it is still a semi-quantitative method. It is reported that the best methods for quantitative measure liver fat content is liver biopsy or magnetic resonance spectroscopy (Dasarathy et al., 2009[7]; Noureddin et al., 2013[28]; Shannon et al., 2011[36]). Fourth, we didn't assess the relation between the circulating VEGF-B and the VEGF-B protein or gene expression in liver tissue. 

In conclusion, our study is the first study to assess the plasma VEGF-B in NAFLD individuals and reveals that VEGF-B concentrations are independently associated with NAFLD. In addition, our result indicates that plasma VEGF-B might be a considerable biomarker for the early detection of hypertension and kidney dysfunction in the general public.

## Notes

Xiaofeng Ye and Wen Kong contributed equally as first authors.

## Funding

This study was supported by National Natural Science Foundation of China (Grant Number 81770843) and the National Key R&D Program of China Grants 2016YFC0901200 and 2016YFC0901203 from the Ministry of Science and Technology.

## Conflict of interest

The authors declare that they have no conflict of interest.

## Authors' contributions

All authors contributed to the study conception and design. Material preparation, data collection and analysis were performed by Xiaofeng Ye, Mohammad Ishraq Zafar, Junchao Zeng, Rui Yang and Wen Kong. The first draft of the manuscript was written by Xiaofeng Ye, Wen Kong and Lu-Lu Chen and all authors commented on previous versions of the manuscript. All authors read and approved the final manuscript.

## Acknowledgements

The authors thank all doctors, nurses, and research staff at the Healthcare Center, Union Hospital, Tongji Medical College, Huazhong University of Science and Technology for their participation in this study.

## Supplementary Material

Supplementary material

Supplementary data 1

Supplementary data 2

## Figures and Tables

**Table 1:**
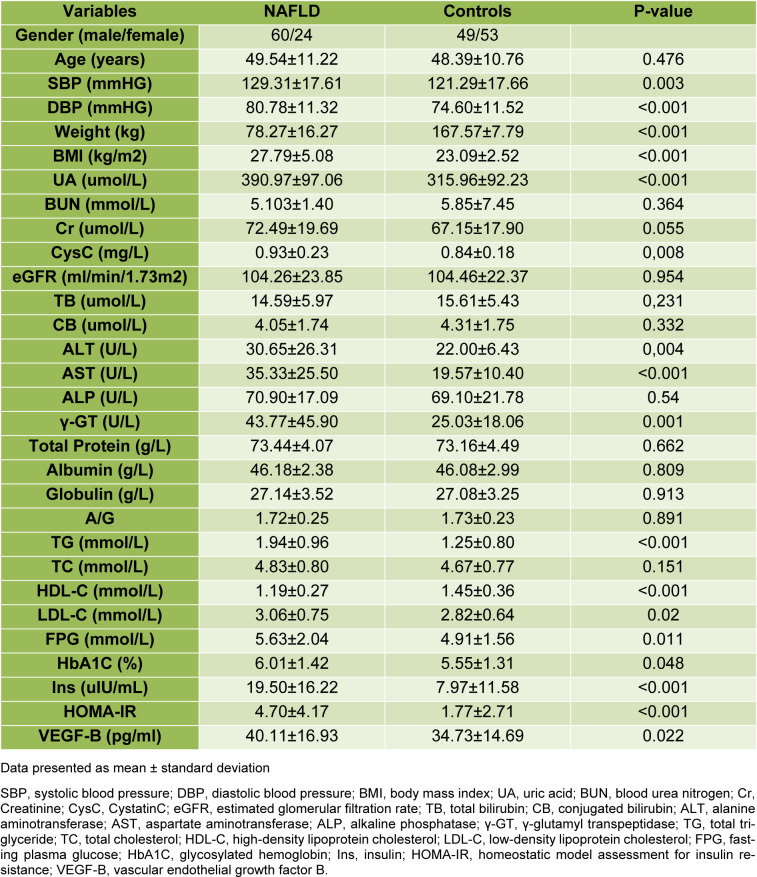
Main characteristics of the NAFLD group and controls

**Table 2:**
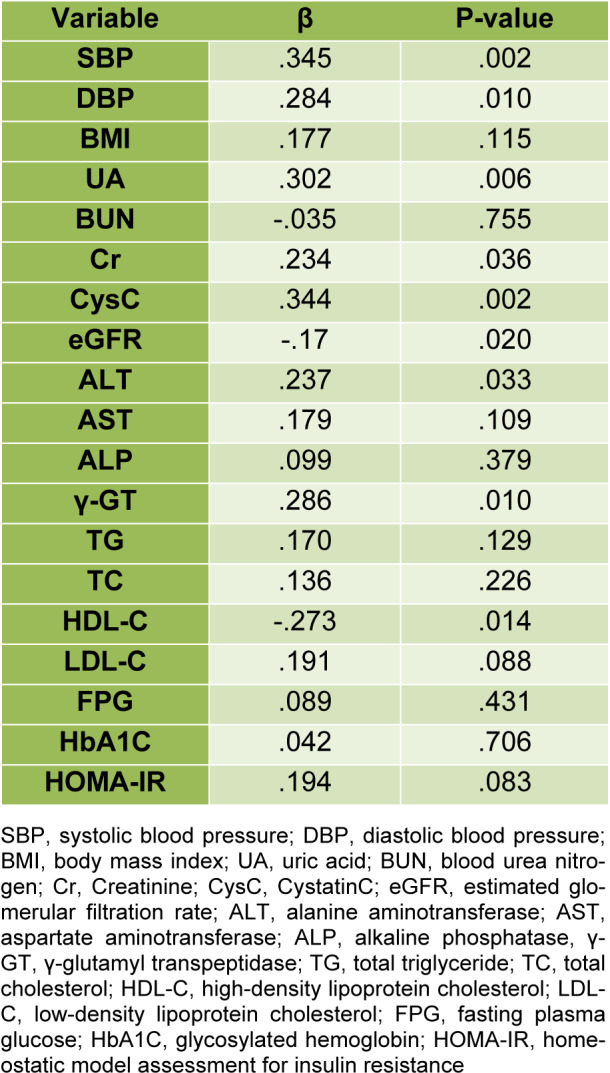
Age and gender-adjusted multivariate analysis for the association of VEGF-B with selected variables
