# Supplementary material for: Plasma vascular endothelial growth factor B is elevated in non-alcoholic fatty liver disease patients and associated with blood pressure and renal dysfunction
Source: EXCLI J. 2020 Aug 20;19:1186–95. doi: 10.17179/excli2020-2647 (PMC7783472; doi:10.17179/excli2020-2647)
Supplement: Supplementary material [file EXCLI-19-1186-s-001.pdf]

EXCLI Journal 2020;19:1186-1195 – ISSN 1611-2156
Received: July 13, 2020, accepted: August 17, 2020, published: August 20, 2020

Supplementary material to:

PLASMA VASCULAR ENDOTHELIAL GROWTH FACTOR B IS ELEVATED IN NON-ALCOHOLIC FATTY LIVER DISEASE PATIENTS AND ASSOCIATED WITH BLOOD PRESSURE AND RENAL DYSFUNCTION

Xiaofeng Ye[1,2#], Wen Kong[1,2#], Mohammad Ishraq Zafar[3], Junchao Zeng[4], Rui Yang[4], Lu-Lu Chen[1,2,*]

[1] Department of Endocrinology, Union Hospital, Tongji Medical College, Huazhong University of Science and Technology, Wuhan 430022, China
[2] Hubei provincial Clinical Research Center for Diabetes and Metabolic Disorders, Wuhan 430022, China
[3] Institute of Reproductive Health, Tongji Medical College, Huazhong University of Science and Technology, Wuhan 430030, China
[4] Healthcare Center, Union Hospital, Tongji Medical College, Huazhong University of Science and Technology, Wuhan 430022, China

[#] Xiaofeng Ye and Wen Kong contributed equally to the manuscript.

[*] **Corresponding author:** Lu-Lu Chen, M.D., Ph.D., Department of Endocrinology, Union Hospital, Tongji Medical College, Huazhong University of Science and Technology, Wuhan 430022, China. Tel.: +86-02785726799; E-mail: cheria_chen@126.com

http://dx.doi.org/10.17179/excli2020-2647

This is an Open Access article distributed under the terms of the Creative Commons Attribution License (http://creativecommons.org/licenses/by/4.0/).

EXCLI Journal 2020;19:1186-1195 – ISSN 1611-2156
Received: July 13, 2020, accepted: August 17, 2020, published: August 20, 2020

Supplementary Table 1: Univariate analysis of variables associated with circulating VEGF-B levels in the study population

Variable	β	P-value
age	0.044	0.548
SBP	0.190	0.009
DBP	0.294	0.000
Weight	0.162	0.025
BMI	0.122	0.091
UA	0.301	0.000
BUN	-0.044	0.548
Cr	0.245	0.001
CysC	0.352	0.000
eGFR	-0.185	0.010
TB	-0.067	0.363
CB	-0.032	0.660
ALT	0.070	0.338
AST	0.104	0.155
ALP	0.083	0.260
γ-GT	0.242	0.001
Total Protein	-0.075	0.310
Albumin	-0.170	0.020
Globulin	0.037	0.617
A/G	-0.117	0.111
TG	0.198	0.006
TC	0.063	0.382
HDL-C	-0.223	0.002
LDL-C	0.102	0.159
FPG	0.051	0.495
HbA1C	0.089	0.282
Ins	0.140	0.054
HOMA-IR	0.174	0.019

SBP, systolic blood pressure; DBP, diastolic blood pressure; BMI, body mass index; UA, uric acid; BUN, blood urea nitrogen; Cr, Creatinine; CysC, CystatinC; eGFR, estimated glomerular filtration rate; TB, total bilirubin; CB, conjugated bilirubin; ALT, alanine aminotransferase; AST, aspartate aminotransferase; ALP, alkaline phosphatase; γ-GT, γ-glutamyl transpeptidase; TG, total triglyceride; TC, total cholesterol; HDL-C, high-density lipoprotein cholesterol; LDL-C, low-density lipoprotein cholesterol; FPG, fasting plasma glucose; HbA1C, glycosylated hemoglobin; Ins, insulin; HOMA-IR, homeostatic model assessment for insulin resistance
